# Muscle Relaxation of the Foot Reduces Corticospinal Excitability of Hand Muscles and Enhances Intracortical Inhibition

**DOI:** 10.3389/fnhum.2016.00218

**Published:** 2016-05-10

**Authors:** Kouki Kato, Tetsuro Muraoka, Nobuaki Mizuguchi, Kento Nakagawa, Hiroki Nakata, Kazuyuki Kanosue

**Affiliations:** ^1^Faculty of Sport Sciences, Waseda UniversitySaitama, Japan; ^2^Japan Society for the Promotion of ScienceTokyo, Japan; ^3^College of Economics, Nihon UniversityTokyo, Japan; ^4^Faculty of Human Life and Environment, Nara Women’s UniversityNara, Japan

**Keywords:** TMS, MEP, SICI, coordination, ipsilateral

## Abstract

The object of this study was to clarify the effects of foot muscle relaxation on activity in the primary motor cortex (M1) of the hand area. Subjects were asked to volitionally relax the right foot from sustained contraction of either the dorsiflexor (tibialis anterior; TA relaxation) or plantarflexor (soleus; SOL relaxation) in response to an auditory stimulus. Single- and paired-pulse transcranial magnetic stimulation (TMS) was delivered to the hand area of the left M1 at different time intervals before and after the onset of TA or SOL relaxation. Motor evoked potentials (MEPs) were recorded from the right extensor carpi radialis (ECR) and flexor carpi radialis (FCR). MEP amplitudes of ECR and FCR caused by single-pulse TMS temporarily decreased after TA and SOL relaxation onset, respectively, as compared with those of the resting control. Furthermore, short-interval intracortical inhibition (SICI) of ECR evaluated with paired-pulse TMS temporarily increased after TA relaxation onset. Our findings indicate that muscle relaxation of the dorsiflexor reduced corticospinal excitability of the ipsilateral hand muscles. This is most likely caused by an increase in intracortical inhibition.

## Introduction

In daily life and sports, many occasions require the simultaneous use of multiple limbs. In some cases, activity in a muscle of one limb interferes with the activity of other muscles in other limbs. For example, when cyclic movements of the ipsilateral upper and lower limbs are executed, movement in one of these limbs is affected by movement of the other (Baldissera et al., 1982; Kelso and Jeka, 1992; Carson et al., 1995; Muraoka et al., 2013). Studies on the underlying neural mechanisms report that muscle contraction in one limb induces an increase in the amplitude of the H-reflex as well as in the excitability of the primary motor cortex (M1) related to the other limb (Jendrássik, 1883; Delwaide and Toulouse, 1981; Hortobágyi et al., 2003; Tazoe et al., 2009; Muraoka et al., 2015). Such interlimb interactions are described as “remote effects”. During cyclic movement of the foot, corticospinal excitability of the resting extensor carpi radialis (ECR) with pronated forearm is higher during dorsiflexion than during plantar flexion. On the other hand, corticospinal excitability of the flexor carpi radialis (FCR) is higher during plantarflexion than during dorsiflexion (Baldissera et al., 2002; Byblow et al., 2007).

It is important to be aware that in our daily activities both muscle contraction and *relaxation* are critical. The necessity for rapid interaction between these two states has been noted to be particularly important for fast paced activities involved in sports (Sakurai and Ohtsuki, 2000) and music performance (Fujii et al., 2009; Yoshie et al., 2009). Furthermore, lack of appropriate muscle relaxation is a typical symptom for patients exhibiting Parkinsonism (Gauggel et al., 2004), dystonia (Yazawa et al., 1999) or stroke (Kamper et al., 2003). We have recently shown that muscle relaxation can influence on remote muscle activities. Muscle relaxation of the foot suppressed electromyographic activity (EMG) of ipsilateral hand muscles that were to simultaneously contract (Kato et al., 2014, 2015). This indicates that muscle relaxation can temporarily produce an inhibitory effect on muscle activity in a different limb. However, the neural mechanisms of inter-limb interactions involved with muscle relaxation are poorly understood.

Neuroimaging and neurophysiological studies using functional magnetic resonance imaging, electroence-phalography, and magnetoencephalography demonstrate that specific brain regions such as the M1 and supplementary motor area (SMA) are activated during muscle relaxation as well as during contraction (Terada et al., 1995, 1999; Toma et al., 1999, 2000; Labyt et al., 2006; Spraker et al., 2009; Wasaka et al., 2012). Thus, muscle relaxation is not just the end of contraction, but rather an active process requiring cortical processing and input. Utilizing single transcranial magnetic stimulation (TMS), Buccolieri et al. (2004) demonstrated that the motor-evoked potential (MEP) amplitude for hand muscles being relaxed from contraction decreased at about same time as the EMG offset. This suggests that excitability of the corticospinal tract controlling the relaxing muscle itself was suppressed during muscle relaxation (Buccolieri et al., 2004). Furthermore, previous studies utilizing paired-pulse TMS showed that intracortical inhibition was activated just before muscle relaxation of the target muscle itself (Buccolieri et al., 2004; Motawar et al., 2012).

In the present study, we utilized TMS to examine the neural mechanisms of the remote effect and its time-course during muscle relaxation. In Experiment 1, we investigated the effects of foot muscle relaxation on corticospinal excitability of a resting hand muscle utilizing single-pulse TMS. We utilized the same experimental protocol as in our previous studies (Kato et al., 2014, 2015). In Experiment 2, we elucidated intracortical inhibitory circuits utilizing the paired-pulse TMS technique. Based on the results of Experiment 2, in Experiment 3 inhibitory mechanisms were carefully investigated by adjusting the MEP amplitude of the test-pulse. We hypothesized that muscle relaxation of the foot muscles would enhance intracortical inhibition and thus suppress corticospinal excitability of the hand muscles.

## Materials and Methods

### Subjects

Experiments 1, 2 and 3 had 11 subjects (mean ± SD = 22.5 ± 2.1 years old, 2 females and 9 males), 10 subjects (mean ± SD = 22.8 ± 1.7 years old, 2 females and 8 males), and ten subjects (mean ± SD = 21.9 ± 1.9 years old, 10 males) respectively. The four subjects who participated in Experiment 2 also participated in the Experiment 3. All were right-handed according to the Edinburgh Inventory (Oldfield, 1971). None of the subjects had a history of neurological or psychiatric disorders. All subjects were fully informed about the purpose of the study and its procedures. Written informed consent was obtained from all subjects. The study was approved by the Ethical Committee of Waseda University. The experiments were conducted in accordance with the Declaration of Helsinki.

### Recordings

Surface EMGs were recorded from the right ECR, FCR, tibialis anterior (TA), and Soleus (SOL) via disposable Ag-AgCl electrodes (1 cm diameter) which were placed over the belly of the muscles. Before the electrodes were attached, the involved area of skin was shaved and treated with alcohol to reduce inter-electrode impedance. Inter-electrode impedance and EMG signals for the four muscles were checked after placing the electrodes. The EMG signal was amplified (MEB-2216, Nihonkoden, Japan) and bandpass filtered between 5 and 1500 Hz. All signals were converted into digital data via an A/D converter system which sampled at a rate of 4000 Hz. We recorded the angle of the right ankle with a goniometer (SG150, Biometrics Ltd., UK).

### Transcranial Magnetic Stimulation (TMS)

TMS was applied to M1 of the left hemisphere using a magnetic stimulator (Magstim 200, Magstim Ltd, UK) connected to a figure-eight coil (110 mm diameter). The subjects wore a tight fitting swimming cap on which the position for stimulation was marked. The coil was held by hand, and its position with respect to the marks carefully monitored. The TMS pulse was delivered to the M1 site at the best location for eliciting MEPs in both the right ECR and FCR (combined hot-spot, Kaneko et al., 1996; Byblow et al., 2007) with a maximum intensity of 1.3 T. The resting motor threshold (rMT) was defined as the minimum stimulus intensity that produced an MEP amplitude with a magnitude greater than 50 μV for both the ECR and FCR for at least 5 out of 10 stimulation trials (Rossini et al., 1994). To exclude the possibility that the strong test stimulation would spread to the foot area, we carefully checked that a cortical silent period was not observed in EMG activity of the foot dorsiflexor or planterflexor before relaxation onset.

### Experimental Design and Analysis

The subjects sat comfortably in a chair, with the right forearm fixed in a horizontal position on an armrest with the palm facing downward. Throughout the recordings, the subjects were instructed not to activate muscles in either hand or in their left foot. The experimenter confirmed that the right foot of the subject did not touch the ground during task execution. To start, the experimentor instructed the subjects to initiate right foot dorsiflexion or plantarflexion at the maximum dorsiflexed or plantarflexed position, and then, to maintain either right foot dorsiflexion or plantarflexion with a moderate and constant effort. The subjects were also instructed to relax as quickly as possible in response to a single tone presented via an earphone. The tone followed the beat of a metronome. The experimenter told the subject not to contract antagonistic muscle of the relaxing foot (e.g., TA while relaxing SOL). The maintenance period for dorsiflexion/plantarflexion before the auditory stimulus was varied randomly from 2 to 5 s. The session during which the subject was asked to relax from the maximum dorsiflexed position was named “TA (tibialis anterior) relaxation session”, because the TA muscle was relaxed in this condition. The session during which the subject was asked to relax from the maximum plantarflexed position was termed the “SOL relaxation session”, because the SOL muscle was relaxed in this session. These two types of relaxation were performed on different days. For practice, the subjects performed relaxation from the dorsiflexed/plantarflexed position at least 10 times before the actual experiments were initiated. If the experimenter noticed unexpected EMG activity in the antagonist of the relaxed muscle (SOL or TA), the practice session was extended until such EMG activity disappeared. To avoid fatigue, the intertrial interval was always more than 10 s. The subjects took a break every 20–30 min. Each experiment lasted approximately 2 h.

The root mean square (RMS) of the relaxing TA or SOL in the period of 0–100 ms before the auditory stimulus was obtained. This activity occurred during the maximum voluntary contraction (%MVC; Table 1) of the other muscles. The obtained value was used as a standard from which to evaluate elicited changes in the degree of relaxation. Before and after all the trials, the MVC for each muscle was measured. For the MVC of TA and SOL, subjects were instructed to develop a force as hard as possible for 3 s, and verbally encouraged to achieve maximum force at the designated joint angles. The subjects performed MVCs of isometric plantar flexion at an ankle joint angle of 90° (i.e., anatomical position) for SOL and dorsiflexion at 90° for TA. An MVC value was determined as the highest mean EMG amplitude observed during the MVC task, within a 1000 ms window. EMG relaxation onset of dorsiflexor or plantarflexor in each trial was visually determined by an experimenter, based on the EMG in the TA or SOL. This method followed that of previous studies (Buccolieri et al., 2004; Begum et al., 2005).

**Table 1:** **EMG activity, EMG latency and Angle latency**.

	Experiment 1	Experiment 2	Experiment 3
**EMG activity of muscles to be relaxed**
TA	43.4 ± 9.9	44.2 ± 12.7	41.8 ± 8.7
SOL	40.2 ± 6.5	39.7 ± 11.0	40.8 ± 10.1
Each value was the root mean square in the 100 ms period just before auditory stimulus (%MVC)
**EMG Latency of relaxation onset (ms)**
TA	163 ± 32	155 ± 32	159 ± 35
SOL	168 ± 35	166 ± 28	171 ± 32
**Angle Latency of relaxation onset (ms)**
TA	221 ± 43	217 ± 44	220 ± 45
SOL	230 ± 47	225 ± 42	231 ± 47

*TA (tibialis anterior) relaxation session; SOL (soleus) relaxation session. Data were expressed as the mean ± SD*.

To determine the latency of right ankle relaxation, Angle latency (Table 1) was defined as the time required for the joint angle to change 0.5° from its baseline position (in the 100 ms period before the auditory stimulus). This method followed that used in a previous study (Kato et al., 2014).

#### Experiment 1

**Figure 1:**
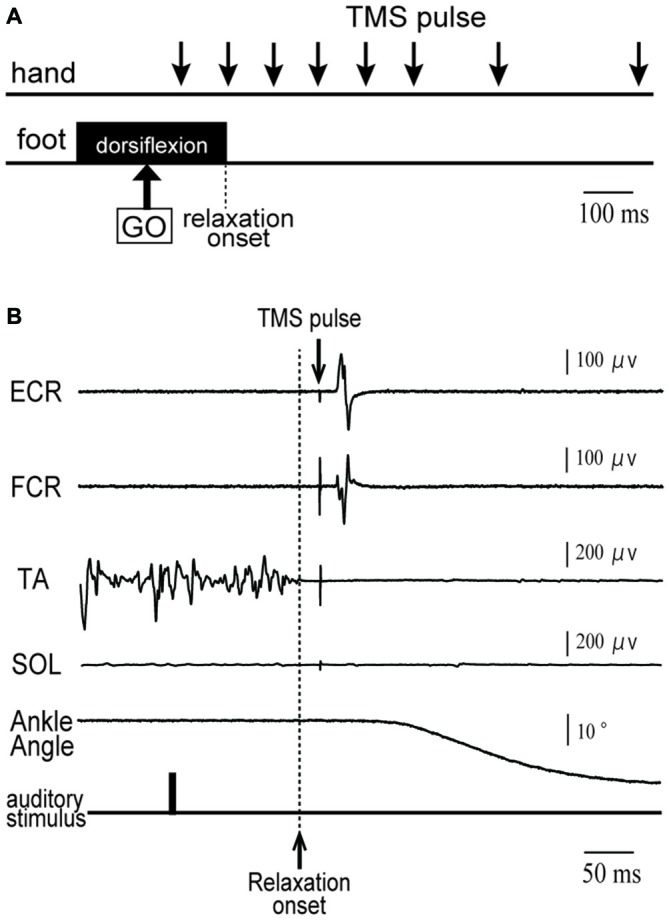
**(A)** Schematic diagram of the timing of transcranial magnetic stimulation (TMS) application in a tibialis anterior (TA) relaxation session. Subjects were asked to maintain right foot dorsiflexion, and to relax as quickly as possible after an auditory stimulus (indicated by GO). A TMS pulse was given over the hand area of the left primary motor cortex (M1) at eight different timings (downward arrows) before and after relaxation onset. **(B)** An example of electromyographic activity (EMG) activity and ankle angle during a single trial for a representative subject. See the text for a detailed explanation.

**Figure 2:**
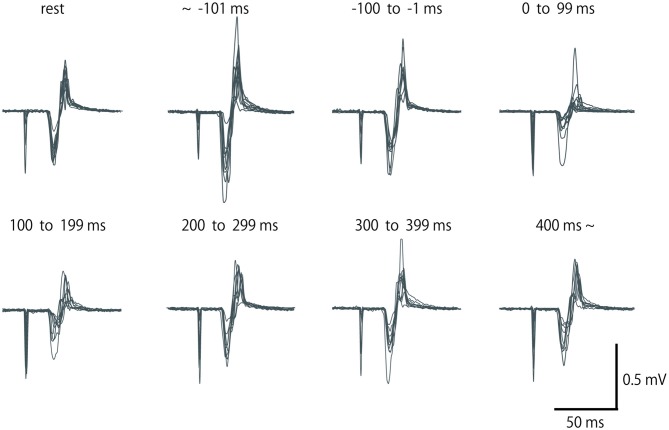
**Row motor-evoked potential (MEP) waveform of extensor carpi radialis (ECR), which was elicited by single-pulse TMS in a subject for eight periods.** Fifteen waveforms in the same period are overlapped. No data were excluded from these overlaps.

During volitional muscle relaxation of the right foot, a single-pulse of TMS over the left M1 was given at the right hand cortical area with one of eight different intervals after the auditory stimulus (50, 150, 250, 350, 450, 550, 750 and 1000 ms; Figures 1A,B). For each subject, a total 135 trials were performed. These trials consisted of 120 trials (8 intervals × 15 trials) with foot relaxation and 15 control trials without foot motion. The trials were performed for both TA relaxation and SOL relaxation sessions. The stimulus intensity for TMS was set at 120% of the rMT. The mean TMS intensity (± standard deviation, SD) used in Experiment 1 was 68 ± 10% of the maximum output of the stimulator. The TMS timing, assessed for the interval from relaxation onset to stimulation for each trial, was grouped into 100 ms bins. To evaluate corticospinal excitability, peak-to-peak MEP amplitudes were recorded. Then, the averaged values of MEP amplitudes in the ECR and FCR were calculated for the following seven periods: before −101 ms; −100 to −1 ms; 0–99 ms; 100–199 ms; 200–299 ms; 300–399 ms; and after 400 ms from relaxation onset (Figure 2). The background EMGs of the ECR and FCR were calculated as the RMS values of the EMGs for a 50 ms window just before the TMS. Trials with a background EMG activity of ECR and FCR that were greater than 25 μV were eliminated from the analysis as error trials. If trials involved definite activity in the antagonist (SOL or TA) greater than 100 μV, data from that trial were also excluded from the analysis (Kato et al., 2015). The mean percentage rate of data rejection was 4.4 ± 1.9% for antagonist activity, 1.0 ± 0.5% for the background EMG of the ECR, and 2.0 ± 1.2% for the background EMG of the FCR, respectively.

#### Experiment 2

To quantitatively evaluate the involvement of intracortical inhibition in the remote effect on relaxation, paired-pulse TMS was utilized over the left M1. Before and after volitional muscle relaxation of the right foot, TMS was given at one of seven different intervals (50, 150, 250, 350, 450, 600 and 900 ms) after the auditory stimulus in both the single- and paired-pulse conditions. We chose the time intervals to lessen the overall time required. A total of 160 trials, composed of 140 trials with 70 single- and 70 paired-pulse TMS, 10 control trials with single-pulse TMS, and 10 control trials with paired-pulse TMS, were performed on each subject. In the paired-pulse TMS condition, the subthreshold conditioning stimulus was delivered 2 ms prior to the suprathreshold test stimulus (Kujirai et al., 1993). Stimulus intensity for the conditioning pulse was set at 80% of the rMT, which would maximally suppress the test MEP amplitude of ECR and FCR at rest by activating inhibitory interneurones (Stinear and Byblow, 2004). The test stimulus intensity was set so that the average peak-to-peak MEP amplitudes in the resting ECR and FCR muscle would be in the range of 0.3–0.5 mV for 10 trials. Single-pulse and paired-pulse TMS were randomly given. Mean conditioning and test stimulus intensities across all subjects were 51 ± 8% and 82 ± 9% of the maximum output of the stimulator.

To calculate intracortical inhibition, we measured the peak-to-peak MEP amplitude elicited by a single-pulse TMS (nonconditioned MEP) and that elicited by a paired-pulse TMS (conditioned MEP). Next, the magnitude of short-interval intracortical inhibition (SICI) was determined following equation of Coxon et al. (2006):

(1) $$SICI\;=\;100\times \left(1-\frac{\text{conditioned}\;\text{MEP}}{\text{nonconditioned}\;\text{MEP}}\right)$$

Then, the averaged values of SICIs in ECR and FCR were calculated for the following seven periods: before −101 ms; −100 to −1 ms; 0–99 ms; 100–199 ms; 200–299 ms; 300–399 ms; and after 400 ms from relaxation onset.

As was done in Experiment 1, trials were eliminated from the analysis in which EMG activity in the antagonist, ECR or FCR was observed. The percentage of elimination for each of the three groups was 1.7 ± 0.9%, 1.4 ± 0.8%, and 0.7 ± 0.4%, respectively.

#### Experiment 3

Based on the results of Experiment 1 and 2, we performed Experiment 3 to further clarify the inhibitory mechanisms in the hand area of M1 that are associated with foot relaxation. For TA relaxation, decreases in corticospinal excitability and increases in SICI were observed in ECR. For SOL relaxation, we observed decreases in corticospinal excitability of FCR, but no significant changes in SICI. We carefully analyzed the SICI of ECR during TA relaxation, and the SICI of FCR during SOL relaxation in the 0–99 ms time window by adjusting the TMS of test-pulse so that the resulting MEP amplitude was the same as that of the rest condition. Two levels of TMS intensity were used as test-pulses in both the TA and SOL relaxation sessions; one (non-adjusted TMS) was the same protocol as that used in Experiment 2, and the other (adjusted TMS) utilized a higher intensity (110–120% of the non-adjusted TMS intensity) as compared with the non-adjusted TMS. This was done in order to induce the same MEP amplitude as that of the control trial (Sanger et al., 2001). This higher intensity compensated for the MEP reduction following muscle relaxation of the foot. The optimal intensity of the adjusted test TMS was decided in the beginning of Experiment 3 for each subject. In this manner the test MEP amplitude that resulted was the same as that of the control trial (Sanger et al., 2001). The mean conditioning, non-adjusted test stimulus, and the adjusted stimulus intensity across all subjects were 53 ± 8%, 79 ± 9%, and 90 ± 9% of the maximum output of the stimulator. For the SICI of ECR during the TA relaxation session, non-adjusted and adjusted TMSs were given 210 ms after the auditory stimulus. This was because notable changes in MEP amplitude and SICI in Experiments 1 and 2 were observed for the period from 0 to 99 ms after relaxation onset, and the latency of relaxation onset was approximately 160 ms after the auditory stimulus. Likewise, for the SICI of FCR during the SOL relaxation session, non-adjusted and adjusted TMSs were given 220 ms after the auditory stimulus, because a notable change in MEP amplitude in Experiment 1 was observed in the period from 0 to 99 ms after relaxation onset, and the latency of relaxation onset was approximately 170 ms after the auditory stimulus. As a control, the SICI was analyzed during the resting condition of the foot. For both the non-adjusted and adjusted TMS sessions, we measured 15 MEPs elicited by the test- and paired-pulse.

### Statistical Analysis

To compare the group data across each experiment, a two-factor analysis of variance (ANOVA) with repeated measures was performed for the three experiments (Experiment 1, 2 and 3) and muscles (SOL and TA) and for both EMG activity of the relaxing muscle before the stimulus as well as the EMG latency and Angle latency of relaxation onset. To analyze the time course of change in MEP amplitude (Experiment 1) and SICI (Experiment 2), the normality of the data distributions were initially assessed with the Shapiro-Wilks test. Since the distributions were found to be non-normal (Experiment 1: *p* < 0.001, and Experiment 2: *p* < 0.05), a non-parametric test was utilized to test for significance. MEP amplitudes and SICI for the seven periods (before −101 ms; −100 to −1 ms; 0–99 ms; 100–199 ms; 200–299 ms; 300–399 ms; and after 400 ms) were compared to those in the resting control period with the multiple-comparison Steel’s test. We utilized a Spearman rank correlation to investigate the relationship between change in MEP amplitude stimulated by a single test pulse and change in the SICI stimulated paired pulse of Experiment 2. For Experiment 3, the difference in MEP amplitudes of single-pulse and SICIs among different TMS timings during foot relaxation (non-adjusted and adjusted TMS) was compared with the control task using a paired *t*-test. Statistical significance was set at *p* < 0.05.

## Results

Mean muscle activity before the auditory stimulus and mean EMG latency and Angle latency of relaxation onset across all subjects are represented in Table 1. There were no significant differences across the three experiments.

**Figure 3:**
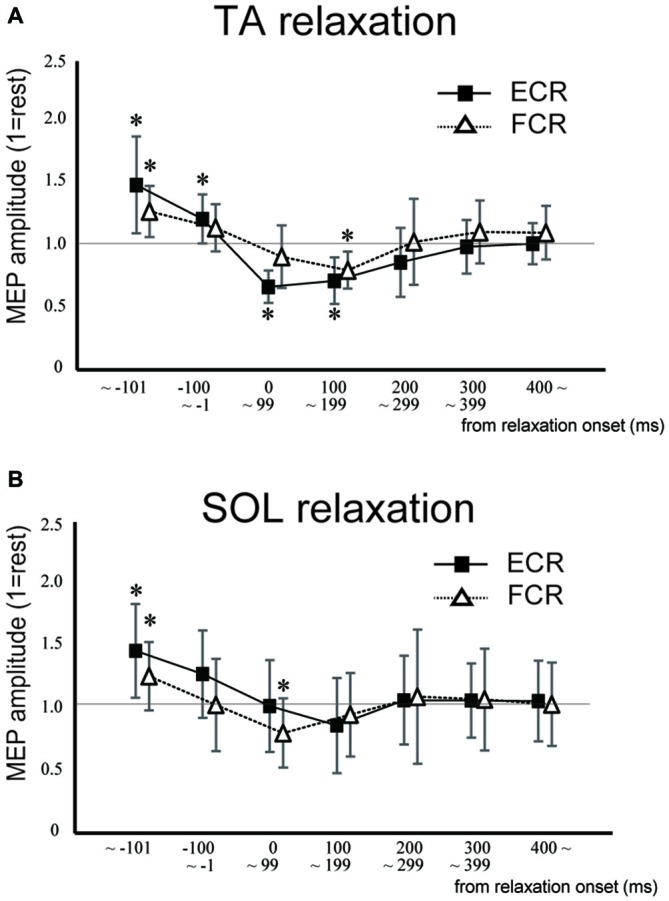
**Mean MEP amplitudes and standard deviation of the ECR (squares) and flexor carpi radialis (FCR; triangles) during TA relaxation (A) and soleus (SOL) relaxation (B) sessions.** Asterisks show statistically significant differences from the resting level (*p* < 0.05).

In Experiment 1, the MEP amplitudes in the ECR were significantly larger in the TA relaxation session than they were in the resting control when TMS was delivered in the periods before −101 ms and −100 to −1 ms (*p* < 0.05 for both; Figure 3A). In contrast, MEP amplitudes in the ECR were significantly smaller in the TA relaxation session than in the resting control when TMS was delivered at 0–99 ms, and 100–199 ms after relaxation onset (*p* < 0.05 for both). The MEP amplitudes in the FCR were significantly larger in the TA relaxation session than in the resting control when TMS was delivered in the period before −101 ms (*p* < 0.05; Figure 3A), and significantly smaller in the TA relaxation session than in the resting control when TMS was delivered at 100–199 ms (*p* < 0.05). MEP amplitudes in the ECR were significantly larger in the SOL relaxation session than in the resting control when TMS was delivered in the periods before −101 ms (*p* < 0.05; Figure 3B). MEP amplitudes in the FCR were significantly larger in the SOL relaxation session than in the resting control when TMS was delivered in the periods before −101 ms (*p* < 0.05; Figure 3B). MEP amplitudes in the FCR were significantly smaller than in the the resting control when TMS was delivered at 0–99 ms after the relaxation onset (*p* < 0.05; Figure 3B).

**Figure 4:**
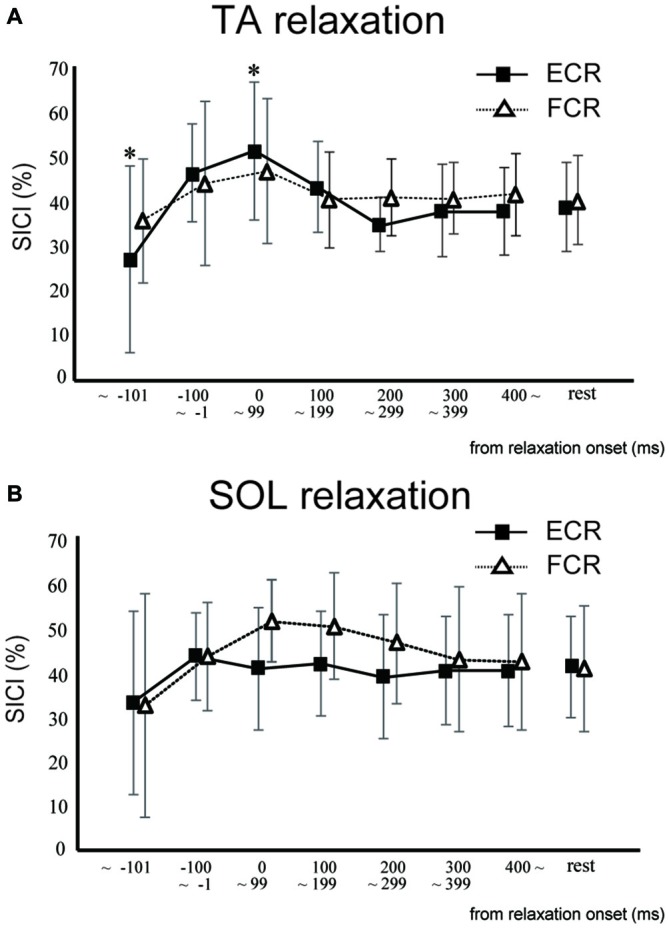
**The mean and standard deviation short-interval intracortical inhibitions (SICIs) of the ECR (squares) and FCR (triangles) during TA relaxation (A) and SOL relaxation (B).** Asterisks depict a statistically significant difference from the resting level (*p* < 0.05).

In Experiment 2, SICI for the ECR was significantly smaller in the TA relaxation session than in the resting control when TMS was delivered in the period before −101 ms (*p* < 0.05; Figure 4A). In contrast, SICI for the ECR was significantly larger in the TA relaxation session than in the resting control when TMS was delivered at 0–99 ms (*p* < 0.05; Figure 4A). There was no significant difference between the SICIs for the ECR and FCR in the SOL relaxation session compared to the resting control when TMS was delivered during any period (Figure 4B). In Experiment 2, SICI increased just after relaxation onset (Figure 4). The Spearman rank correlation analysis revealed a correlation trend between the decrease in MEP and increase in the SICI for the period of 0–99 ms (*r* = −0.618, *p* = 0.057).

**Figure 5:**
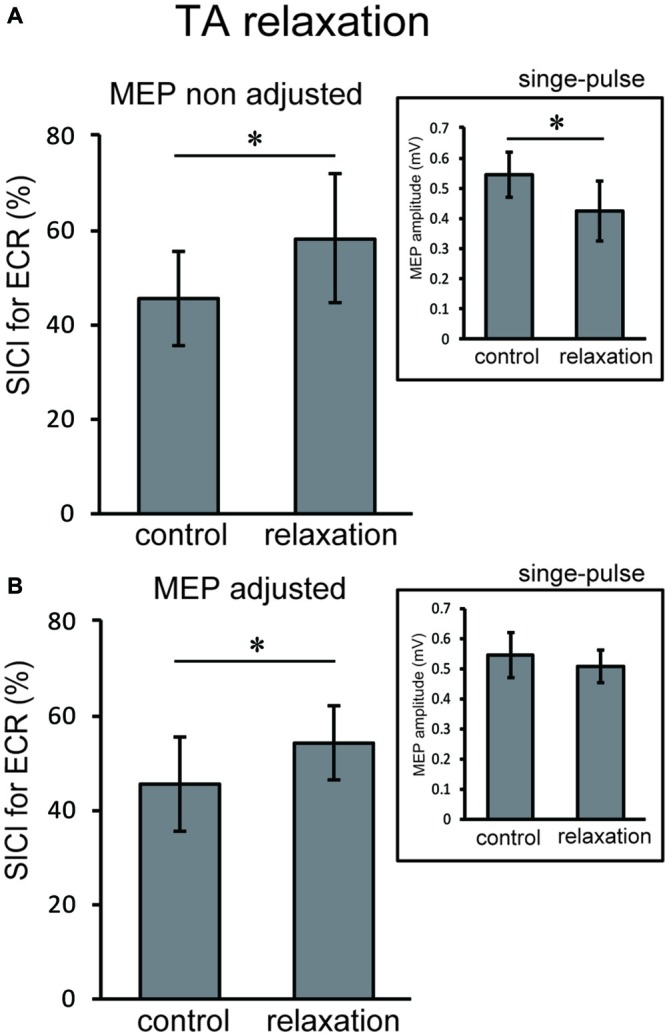
**Using the original test pulse, the SICI after TA relaxation was significantly larger (A).** However, the test MEP was smaller than the control, in correspondence with the results of Experiment 1 (**A**, inset). When the MEP amplitudes were adjusted (**B**, inset), the SICI after TA relaxation (54.00 ± 6.75) was also larger. Error bars indicate SD. Asterisks show statistically significant differences (*p* < 0.05).

**Figure 6:**
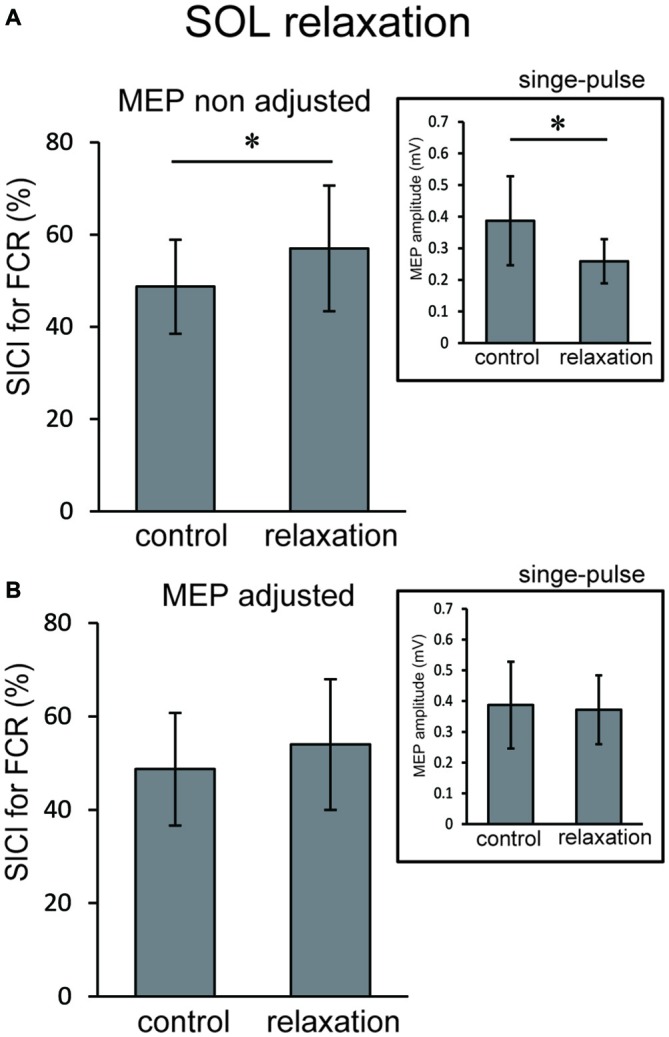
**Using the original test pulse, the SICI after SOL relaxation was significantly larger (A).** However, the test MEP was smaller than the control, in correspondence with the results of Experiment 2 (**A**, inset). When the MEP amplitudes were adjusted (**B**, inset), no difference in the SICI after SOL relaxation was observed **(B)**. Error bars indicate SD. Asterisks show statistically significant differences (*p* < 0.05).

In Experiment 3, using the non-adjusted test pulse, SICI of the ECR after TA relaxation was larger than in the resting condition (Figure 5A). In this case, the test MEP was smaller than the control (*p* < 0.05) as in Experiment 1 (Figure 5A, inset). Even after the MEP amplitudes were adjusted (Figure 5B, inset), SICIs of the ECR after TA relaxation (54.00 ± 6.75%) were still significantly larger than in the resting condition (*p* < 0.05; Figure 5B). For SOL relaxation, when the original test pulse was given, SICI of the FCR after SOL relaxation was larger than in the resting condition (Figure 6A). The test MEP was smaller than for the control (*p* < 0.05; Figure 6A, inset). When MEP amplitudes were adjusted (Figure 6B, inset), the difference in the SICI after SOL relaxation was abolished (*p* = 0.42; Figure 6B).

## Discussion

The objective of this study was to investigate whether the corticospinal tract and intracortical inhibitory systems controlling the forearm muscles were affected by volitional relaxation of the foot on the ipsilateral side. We analyzed the time course of MEP amplitudes and SICIs in the ECR and FCR, based on the onset of TA or SOL relaxation. We found that corticospinal excitability of the ECR decreased in the period from 0–199 ms after relaxation onset of the TA (Figure 3A), as compared with the corresponding resting condition. We also found that SICI of the ECR increased during the period from 0–99 ms after relaxation onset of the TA, as compared with the resting condition (Figures 4A, 5). This effect remained even after the TMS was adjusted so that the test MEP amplitude was the same as in the resting condition (Figure 5). During SOL relaxation, decreases in corticospinal excitability for the period from 100–199 ms were observed in the FCR (Figure 3B). Although an increase in SICI of the FCR in the non-adjusted condition was only observed in Experiment 2 (Figure 4A), we observed no changes in the SICI when the TMS was adjusted as described earlier (Figure 6).

Our present finding that corticospinal excitability of the hand muscles was reduced just after foot relaxation corresponds well with the results of our previous studies (Kato et al., 2014, 2015). In those studies, foot muscle relaxation induced a decrease in the EMG activity of a hand muscle that was to contract simultaneously with foot relaxation. During voluntary muscle relaxation, a decrease in corticospinal excitability of the relaxed muscle itself has been observed in other laboratories (Buccolieri et al., 2004; Begum et al., 2005). Utilizing results obtained with the paired-pulse TMS technique, Buccolieri et al. (2004) and Motawar et al. (2012) suggested that the decreased corticospinal excitability might have been caused by enhanced intracortical inhibition. Likewise, in the present study, SICI for the hand muscles was increased just after relaxation onset of the foot dorsiflexor. However, this was observed only for the extensor (Figures 5, 6). Thus, at least for the hand extensor, changes in intracortical inhibition might cause the decrease in corticospinal excitability seen during volitional muscle relaxation not only in the relaxed muscles themselves (Buccolieri et al., 2004; Motawar et al., 2012), but also in other muscles of the remote limb.

With respect to the time-course of changes in MEP amplitudes and SICIs, the suppression of corticospinal excitability and enhancement of intracortical inhibition compared to the resting condition were observed “after” relaxation onset. On the other hand, it has been previously reported that intracortical inhibition of a contracted muscle itself increased just “before” its relaxation onset (Buccolieri et al., 2004; Motawar et al., 2012). It seems that the inhibitory process involving remote muscles is activated slightly after the inhibitory process of the relaxed muscle itself. We previously reported that EMG activity and force magnitude of the hand extensor decreased in the period from 0 to 200 ms “after” relaxation onset of the foot (Kato et al., 2015). This result corresponds well with the time-course of intracortical inhibition in the present study. For the ECR, moreover, while an increase in SICI was observed in the period from 0 to 99 ms after TA relaxation onset, a decrease in corticospinal excitability was observed in the period from 0 to 199 ms. Thus, the end point of change in the SICI is preceded by that of corticospinal excitability during TA relaxation. Buccolieri et al. (2004) showed that an increase in SICI occurred prior to the onset of MEP decrease. They also noted that “increased cortical inhibition may play a role in suppressing corticospinal excitability during relaxation.” Furthermore, we observed a nearly significant correlation between the decrease in MEP and increase in the SICI for the period 0–99 ms. Therefore, our findings indicate that changes in SICI might play an important role in suppressing corticospinal excitability of the hand muscle during foot relaxation.

The Go/No-go task has been widely utilized to investigate the inhibitory processes of motor execution (Waldvogel et al., 2000; Nakata et al., 2006). In the Go/No-go task, subjects are required to respond to one cue (the Go stimulus), and not respond to another cue (the No-go stimulus). During the No-go portion of the task, corticospinal excitability decreased as compared to the resting condition (Hoshiyama et al., 1996, 1997; Leocani et al., 2000; Waldvogel et al., 2000; Yamanaka et al., 2002; Nakata et al., 2006). Furthermore, the reduction of corticospinal excitability for the remote upper limb was observed during the No-go cue for the foot (Badry et al., 2009). This study also demonstrated that a reduction in corticospinal excitability for a hand muscle was observed with a peak latency at 400 ms after the cue for the No-go trial that involved the ipsilateral foot (Badry et al., 2009). For the target muscle itself, corticospinal excitability during the No-go portion of the Go/No-go task decreased approximately 200 ms after the No-go cue signal (Yamanaka et al., 2002). Thus, the inhibitory process involving the remote muscle during the No-go portion of the task is activated later than the inhibitory process of the target muscle itself. While the No-go portion of the task involves the suppression of a planned action and relaxation is the cessation of ongoing contraction, both situations might involve a similar effect of inhibition on the remote muscles.

Previous fMRI studies have shown that relaxation of muscles in a single limb produces activation in various brain regions including the M1, SMA, and pre-SMA (Toma et al., 1999; Oga et al., 2002). Although the current study showed a decrease in corticospinal excitability and an increase in intracortical inhibitory circuit for the hand area of M1 during foot muscle relaxation, it is still unknown as to which brain regions and neural pathways are responsible for this effect. Since there are no anatomical connections between hand and foot areas in M1 (Huntley and Jones, 1991; Brown et al., 1991), activity in the M1 foot area involved with foot relaxation would not directly affect activity in the M1 hand area. When coordinating movements of the upper and lower limbs, the SMA is suggested to play a substantial role in the distributed motor network for this coordination (Debaere et al., 2001). In addition, the SMA-M1 network contributes to a nonspecific facilitation effect of limb movement on remote muscles (Byblow et al., 2007). It is expected that a future study will elucidate whether the SMA is included in inhibitory remote effects of muscle relaxation. The present results did not show an obvious muscle specificity in the remote effect of foot contraction/relaxation on excitability of the forearm M1. First, before the relaxation (i.e., during contraction) of both the TA and SOL, we observed significant increases in MEP for both the ECR and FCR (Figure 3). This corresponds well with the results in previous studies which indicate that the facilitatory effect of the cortical mechanism on one limb during static contraction of other limbs displays a non-topographic characteristic (Tazoe et al., 2007; Chiou et al., 2013; Tazoe and Komiyama, 2014). The fact that the decrease in corticospinal excitability was observed in both ECR and FCR during TA relaxation indicated that the effect of foot relaxation spread to the forearm muscles, regardless of whether they were extensors or flexors (Figure 3). However, the decrease in corticospinal excitability lasted longer for the ECR, and the increase in SICI was observed only for the ECR (Figure 4). Likewise, SOL relaxation induced a decrease in corticospinal excitability only for the FCR (Figure 3). This suggests that the neural interaction between specific muscles (TA-ECR and SOL-FCR) is stronger than that between other muscle combinations during relaxation of muscles in the lower limb. For cyclic dorsiflexion-plantarflexion of the foot, which includes repetitive contraction and relaxation, changes in corticospinal excitability of the ipsilateral forearm muscles has been shown to be dependent upon whether foot movement is in the dorsiflexion or plantarflexion phase with pronated forearm position (Baldissera et al., 2002; Byblow et al., 2007). Thus, corticospinal activity of the hand extensor/flexor is augmented during foot dorsiflexion/plantarflexion, respectively. These combinations (dorsiflexor-hand extensor and plantarflexor-hand flexor) are similar to those of the present study. Then, during SOL relaxation, why didn’t we observe an increase in SICI for both forearm muscles, especially for the FCR? The effects of foot muscle relaxation on the hand area might be stronger for the TA as compared with the SOL. Indeed, a previous study demonstrated that corticospinal projections to the lower limb motoneurons in humans are stronger to the flexor (TA) than to the extensor (SOL) muscles (Brouwer and Ashby, 1992). However, we need further study to clarify whether the remote effects of muscle relaxation are indeed dependent upon specific muscle couplings.

In conclusion, our findings using TMS indicated that relaxation of foot muscles reduced the corticospinal excitability involved with the control of ipsilateral hand muscles, and enhanced intracortical inhibition of hand extensor muscles.

## Author Contributions

Conceived and designed the experiments: KoK, TM, HN and KaK. Performed the experiments: KoK, NM and KN. Analyzed the data: KoK. Contributed reagents/materials/analysis tools: KoK, TM and KN. Wrote the article: KoK, HN and KaK.

## Conflict of Interest Statement

The authors declare that the research was conducted in the absence of any commercial or financial relationships that could be construed as a potential conflict of interest.
